# Inclusion of joint laxity, recurrent patellar dislocation, and short distal ulnae as a feature of Van Den Ende-Gupta syndrome: a case report

**DOI:** 10.1186/s12881-018-0531-y

**Published:** 2018-01-30

**Authors:** Mohammad M. Al-Qattan, Doaa F. Andejani, Nadia A. Sakati, Khushnooda Ramzan, Faiqa Imtiaz

**Affiliations:** 1 Department of Surgery, King Saud University, PO Box 18097, Riyadh, 11415 Saudi Arabia; 2 King Faisal Specialist Hospital & Research Center, Riyadh, Saudi Arabia; 3 National Hospital, Riyadh, Saudi Arabia; 4 The Saudi Plastic Surgery program, King Fahad Medical City, Riyadh, Saudi Arabia; 5 Department of Pediatrics, King Faisal Specialist Hospital& Research Center, Riyadh, Saudi Arabia; 6 Department of Genetics, King Faisal Specialist Hospital & Research Center, Riyadh, Saudi Arabia

**Keywords:** Van Den Ende-Gupta syndrome, Joint laxity, Patellar dislocation, Short distal ulna

## Abstract

**Background:**

Van Den Ende-Gupta Syndrome (VDEGS) is an extremely rare autosomal recessive syndrome with less than 20 reported families (approximately 40 patients) in the worldwide literature.

**Case presentation:**

We have assessed one consanguineous Saudi family with typical features of VDEGS. Two siblings were affected with almost identical features; including blepharophimosis, arachnodactyly, flexion contractures of the elbows, camptodactyly, slender ribs, hooked lateral clavicular ends, and bilateral radial head dislocations. Both patients had several unusual features; including joint laxity, flat feet, recurrent patellar dislocations, and bilateral short distal ulnae. Full sequencing of *SCARF2* revealed a homozygous mutation c.773G > A (p. Cys258Tyr) in both affected children. The parents (both with no abnormalities) were heterozygous for the same mutation.

**Conclusion:**

Joint laxity, recurrent patellar dislocations, and short distal ulnae should be included as part of the clinical spectrum of VDEGS.

## Background

Van Den Ende-Gupta Syndrome (VDEGS, MIM 600920) is an extremely rare autosomal recessive syndrome with less than 20 reported families (approximately 40 patients) in the worldwide literature [1–7]. Homozygous mutations in *SCARF2* are responsible for the syndrome [5]. The *SCARF2* gene is located at the 22q11.2 region, which contains the critical region of the velo-cardio-facial/Di George syndrome (MIM 18840). Some patients with VDEGS have compound heterozygosity for the common Di George 22q11.2 microdeletion and a hemizygous *SCARF2* splice mutation [6]. VDEGS is characterized by blepharophimosis, hypoplastic maxillae, narrow nose with flat nasal bridge, everted lower lip, triangular face, high-arched palate, arachnodactyly (long, slender digits, which are more pronounced in the thumbs and big toes), multiple joint contractures (camptodactyly, flexion contractures of the elbows, stiffness of the knees), bilateral radial head dislocations, slender ribs, abnormalities of the clavicles (tapered or hooked lateral clavicular ends), valgus deformities of the big toes, and faint/absent distal flexion creases of the fingers. As expected, the clinical features partially overlap with the velo-cardio-facial/Di George syndrome phenotype, such as the bulbous nasal tip, the palatal abnormalities, and the transient hypocalcemia at birth [5, 6]. Several clinical features of VDEGS (specifically the blepharophimosis, arachnodactyly, and multiple joint contractures including the camptodactyly) are also seen in Marden-Walker syndrome (MIM 248700) caused by *PIEZO2* mutations. However, Marden-Walker syndrome is distinguished by the presence of severe mental retardation, hypotonia, and major brain abnormalities, including cerebellar and brainstem hypoplasia [7]. Furthermore, over 80% of patients with Marden-Walker syndrome show kyphoscoliosis and ear anomalies (low-set, dysplastic ears).

In this paper, we report on a Saudi family with two affected children with VDEGS. Both patients had unusual features; including joint laxity, recurrent patellar dislocation, and short distal ulnae. The literature is reviewed to confirm that these features should be included in the clinical spectrum of VDEGS. CARE guidelines were followed in this report.

## Case presentation

**Fig. 1:**
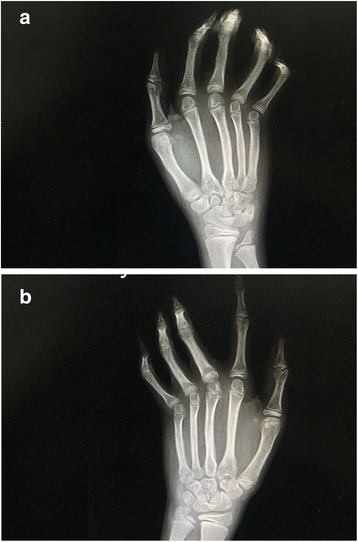
X-Rays of the Right (**a**) and Left (**b**) hands of the male sibling showing the classic arachnodactyly with long, slender bones. Also note the bilateral short distal ulnae with abnormal configuration of the distal ulnar epiphyses

**Fig. 2:**
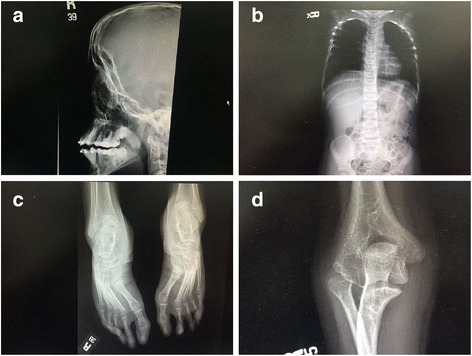
Radiographic images of the classic features of the VDEGS syndrome: **a** the hypoplastic maxillae, **b** the slender ribs with hooked lateral clavicular ends, **c** the valgus deformity of the big toes. Also note the long, slender bones in the feet. **d** Radial head dislocation

**Fig. 3:**
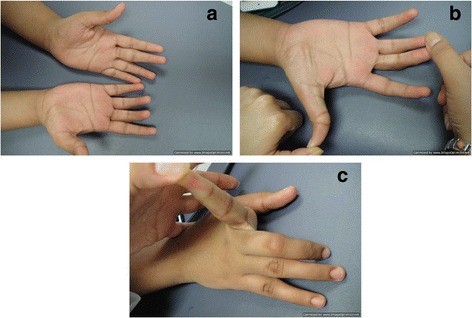
**a** The appearance of the hands of the female sibling showing the camptodactyly (flexion contractures) of the right middle/ring fingers and of the left ring finger. Also note the faint/absent distal flexion creases of multiple fingers. **b** Demonstration of the hyper-extension of the interphalangeal joint of the thumb and the flexion contracture of the middle and ring fingers. **c** Demonstration of hyper-extension of the index finger

We have assessed one consanguineous Saudi family with typical features of VDEGS. The parents were first degree cousins and had no abnormalities. Two of their four children were affected. The affected children were referred to the Hand Surgery Clinic at ages 11 and 15 years for consideration for surgical correction of camptodactyly. The phenotypes of both affected siblings, a male and a female, were almost identical and included blepharophimosis, hypoplastic maxillae, narrow nose with flat nose bridge, everted lower lips, triangular face, high arched palate, arachnodactyly, flexion contractures of the elbows with bilateral radial head dislocations, slender ribs, and hooked lateral clavicular ends (Figs. 1 and 2). Both had bilateral hand camptodactyly: the brother had involvement of the ulnar three fingers on the right and all fingers on the left; and the sister had involvement of the middle and ring fingers on the right and the ring finger on the left. Faint/absent distal flexion creases of multiple fingers were also noted in both patients (Fig. 3a).

**Fig. 4:**
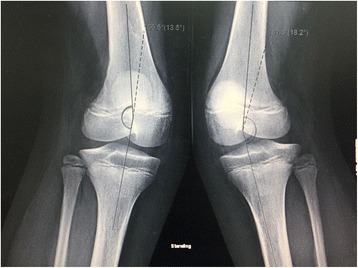
X-Rays of the knees showing genu valgus deformity secondary to ligament laxity

**Fig. 5:**
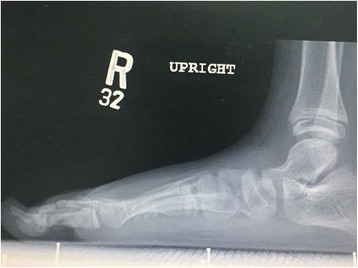
The flat-foot deformity

Besides these typical features, both patients had several unusual features. Generalized joint laxity was demonstrated in multiple joints. The digits of the hands which were not involved with the camptodactyly were hyper-extensible, indicating joint laxity (Fig. 3b, c). Both patients also had laxity of the shoulders and knees with genu valgus deformity (Fig. 4). The feet had severe flat-feet deformities with loss of arches (Fig. 5). Finally, both children suffered from recurrent patellar dislocation and had bilateral short distal ulnae. The short distal ulnae were associated with abnormal configuration of the distal ulnar epiphysis (Fig. 1). Intelligence was normal. An ultrasound of the abdomen showed no abnormalities in both children.

Based on the clinical diagnosis, the coding region of the *SCARF2* gene (NM_153334.4) was amplified using standard conditions (primers and protocol are available on request) and fully sequenced. After an informed consent, sequencing of *SCARF2* revealed a homozygous mutation c.773 G > A (p.Cys258Tyr) in both affected children. The parents were heterozygous carriers for the same mutation. This mutation is known to be disease-causing [5].

## Discussion and conclusion

VDEGS is extremely rare and is worth reporting. However, the main aim of reporting our family was the delineation of the unusual features described above. Almost 100% of patients with VDEGS show flexion contractures of the elbows and camptodactyly of the fingers of the hands [2]. Our patients had these two typical features with very unusual joint laxity in the digits not involved with the camptodactyly as well as laxity of the shoulders and the knees. The severe flat feet may also be considered secondary to ligament laxity. Most striking was the recurrent bilateral patellar dislocations in both affected children. After an extensive review of the literature, we found that recurrent patellar dislocation was seen in one patient reported by Patel et al. [3]. This indicated that joint laxity and patellar dislocation should be included in the VDEGS phenotype.

Ali et al. [2] reviewed the literature and found that 50% of patients with VDEGS suffer from bilateral radial head dislocation. The pathogenesis of congenital radial hand dislocation has recently been reviewed by Al-Qattan et al. [8]. It is interesting to note that the primary pathology leading to radial head dislocation is variable and includes collagen abnormalities (which is associated with ligament laxity) as well as developmental abnormalities of the radial head and disproportionate growth of the radius and ulna.

Another unusual finding in our patients was the bilateral shortness of the distal ulnae. This feature was not mentioned in reviews of VDEGS [2, 3, 9]. However, our literature review revealed that bilateral shortness of the distal ulnae was reported in the original patient of Gupta et al. [10] as well as in the patient reported by Migliavacca et al. [4]. This indicated that shortness of the distal ulnae should also be included in the VDEGS phenotype.

Our review of the literature revealed that the clinical features of VDEGS may be classified into 3 groups (Table 1). “Constant or almost constant” features are seen in 90–100% of reported cases, while “common” features are seen in 40–89% of cases as shown in Table 1 [1–7, 9, 10]. “Uncommon” features are seen in less than 40% of cases and these include small scapulae [2, 5], bowing of the femoral and humeral shafts [2, 3], bowing of the proximal ulna [11], cleft palate [12], 2–3 toe syndactyly [2, 3, 5], hydronephrosis [3], dilatation of the renal pelvis [5], scaphocephaly [5], trigonocephaly [3], speech delay [3], sacral dimple [5], various ear abnormalities [4–7, 11], various eye abnormalities [4, 6], cerebellar enlargement [7], scoliosis [4], transient hypocalcemia at birth [6], hypoplastic nails with short or hypoplastic distal phalanges [10], clinodactyly [10, 11], club feet [7, 12, 13], hypoplasia of the glenoid fossa [3, 10, 13], micrognathia [5, 13], learning disability [7], single umbilical artery [10], laryngeal abnormalities [14], sensorineural hearing loss [1, 9], deviated nasal septum [1, 9], hypospadias [9], atrial septal defect [9], significant developmental delay [9], joint laxity [current report], recurrent patellar dislocation [3, current report], and short distal ulnae [4,10, current report].

**Table 1:** Clinical features of VDEGS

‘Constant’ features (seen in 90–100% of cases)	‘Common’ features (seen in 40–89% of cases)	‘Infrequent’ features (seen in less than 40% of cases)
Blepharophimosis, hypoplastic maxillae, nasal abnormalities (one or more of the following: narrow nose, flat nasal bridge, nasal tip abnormalities, beaked nasal appearance, and occasionally pseudocleft of the columella), everted lower lip, triangular face, arachnodactyly, camptodactyly in the fingers, flexion contracture or limited mobility of the elbows	High arched palate, bilateral radial head dislocation (with or without hypoplasia of the radial head), slender ribs, clavicular abnormalities, valgus deformities of the big toes, faint/absent distal flexion creases of the fingers	Small scapulae, bowing of the femoral and humeral shafts, bowing of the proximal ulna, cleft palate, 2–3 toe syndactyly, renal abnormalities, craniosynostosis, speech delay, sacral dimple, ear abnormalities (low-set ears, posteriorly-rotated ears, folded ear helix, prominent ears, large ears), eye abnormalities (microphthalmia, corneal opacity, nystagmus, squint), cerebellar enlargement, scoliosis, transient hypocalcemia at birth, hypoplastic nails with short distal phalanges, clinodactyly of fingers or toes, club feet, hypoplasia of the glenoid fossa, micrognathia, learning disability, single umbilical artery, laryngeal abnormalities, sensorineural hearing loss, deviated nasal septum, hypospadias, atrial septal defect, significant developmental delay, joint laxity, recurrent patellar dislocation, short distal ulnae.

The SCARF2 protein is a calcium binding protein which is highly expressed during development in the nose, oral epithelium, and ribs [15]. However, this does not fully explain the pathogenesis of most of the clinical features of VDEGS. There is a strong need for knockout animal models in order to thoroughly investigate SCARF2 function. Recently, a canine model of VDEGS with a 2-bp deletion in *SCARF2* leading to a severely truncated protein was identified. Affected animals show a skeletal syndrome with some similar features to the human phenotype, including patellar subluxation [16].

Although Marden-Walker syndrome also show the blepharophimosis, arachnodactyly, and joint contractures in the phenotype, we did not consider this diagnosis because of the lack of its other distinguishing features (mental retardation, hypotonia, and major brain abnormalities) in our cases.

It is important to note that the unaffected siblings did not share any symptoms with the affected siblings. Despite this, one limitation of our study is that we did not do whole exome sequencing. In consanguineous marriages, biallelic or even triallelic mutations in two distinct genes or co-inheriting genetic modifiers may be considered and could be identified with whole exome sequencing. However, the unusual features we report in our paper were also reported by previous authors and the canine model of VDEGS also show patellar subluxation in the phenotype [16].

We conclude that joint laxity, recurrent patellar dislocation, and short distal ulnae should be included in the VDEGS phenotype.

## Abbreviations

VDEGS: Van Den Ende-Gupta Syndrome
